# Balloon technologies for pulmonary vein isolation—12-month outcome and comparison of the novel radiofrequency balloon with the cryoballoon in patients with paroxysmal atrial fibrillation

**DOI:** 10.1007/s00392-024-02401-w

**Published:** 2024-03-13

**Authors:** Jan-Hendrik van den Bruck, Jonas Wörmann, Arian Sultan, Karlo Filipovic, Katharina Seuthe, Susanne Erlhöfer, Cornelia Scheurlen, Sebastian Dittrich, Jan-Hendrik Schipper, Jakob Lüker, Daniel Steven

**Affiliations:** Department of Electrophysiology, Faculty of Medicine and University Hospital Cologne, University of Cologne, Kerpener Strasse 62, 50937 Cologne, Germany

**Keywords:** Atrial fibrillation, Cryoballoon ablation, Heliostar™, Radiofrequency balloon, Single shot pulmonary vein isolation

## Abstract

**Background:**

The cryoballoon (CB) has become a standard tool for pulmonary vein isolation (PVI), but the technology is limited in certain ways. A novel RF-balloon (Heliostar™, Biosense Webster, CA, USA) promises the advantages of a balloon technology in combination with 3D mapping.

**Methods:**

To assess procedural data and outcome, all patients undergoing RF-balloon PVI were included and compared with data from consecutive patients undergoing CB PVI for paroxysmal AF.

**Results:**

A total of 254 patients (63 ± 13 years, 54% male) were included: 30 patients undergoing RF-balloon and 224 patients CB PVI. Baseline parameters were comparable. Procedure duration (104.3 ± 35.3 min vs. 69.9 ± 23.1 min; *p* ≤ 0.001) and fluoroscopy time (16.3 ± 7.1 min vs. 11.6 ± 4.9 min; *p* ≤ 0.001) were longer using the RF-balloon; ablation time (43.5 ± 17.9 vs. 36.4 ± 15.6; *p* = 0.08) did not differ, and time-to-isolation (TTI) was shorter (18.2 ± 7.0 s vs. 62.8 ± 35.1 s; *p* ≤ 0.001). Second-generation RF-balloon cases showed shorter ablation time and TTI at comparable procedure duration and fluoroscopy time. One pericardial effusion occurred with the RF-balloon due to complicated transseptal access. During CB PVI in 4/224 patients (1.8%), a phrenic nerve palsy was observed. After 12 months, 78% of patients after RF-balloon and 81% of patients after CB PVI (*p* = 0.5) were free from atrial arrhythmias.

**Conclusion:**

The RF-balloon was safe and effective. Compared with the CB, TTI was shorter, but procedure durations and fluoroscopy times were longer. This can be attributed to a learning curve and the initial necessity for separate 3D map preparation. Considering the results with the second-generation RF-balloon, more experience is needed to determine the potential benefits.

**Graphical Abstract:**

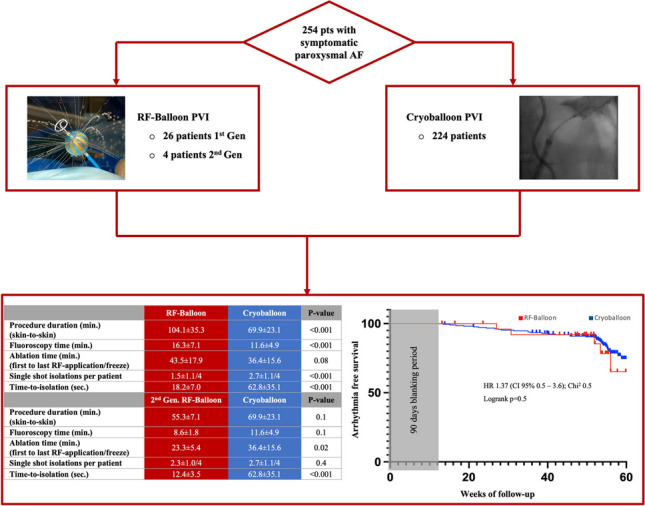

## Introduction

Pulmonary vein isolation (PVI), being more effective at maintaining sinus rhythm than medical therapy, has become the key therapy in the treatment of symptomatic AF [1–3].

By now, there are several types of catheters, ablation techniques, and energy forms available to achieve PVI. While point-by-point radiofrequency (RF) ablation combined with 3D mapping was always considered as the gold standard technology, ultimately the cryoballoon ablation with its relatively easy handling and the shorter procedure durations has emerged as the most frequently used alternative [4]. But besides these advantages, the cryoballoon has several limitations. Not only the higher fluoroscopy burden and contrast agent exposition due to the unavailability of 3D mapping but especially the very restricted possibilities of lesion formation are crucial limiting factors [4, 5]. In this context, a recently introduced multi-electrode RF-balloon catheter (Heliostar™, Biosense Webster, Diamond Bar, CA, USA) intends to combine the advantages of a single-shot balloon technology with integration into a 3D mapping system and the opportunity of more flexible energy delivery. Two pre-market studies had already demonstrated a safe application of the novel RF-balloon catheter with good clinical outcomes at 12 months [6, 7]. Furthermore, the recently published first early market release data could confirm these promising results regarding acute procedural outcomes [8, 9].

But there is still very limited data available regarding the safety and efficacy of the novel RF-balloon, and most notably, there is, to the best of our knowledge, no data available comparing the RF-balloon to the established single-shot technique and also regarding long-term procedural outcome. We therefore aimed to assess procedure characteristics, efficacy, and safety of the early market release RF-balloon application in direct comparison with the established cryoballoon.

## Methods

### Inclusion criteria and study population

All patients undergoing RF-balloon-based PVI between September 2021 and November 2022 at the University Hospital of Cologne were prospectively included in this single-center registry. Inclusion criteria were symptomatic paroxysmal AF, age > 18 years, and prior written informed consent. Consecutive patients were included, and there were no predefined criteria for RF or cryoballoon use. Patients with prior left atrial ablation, persistent AF, or history of atrial tachycardia (AT) or atrial flutter were excluded. Procedural data and outcome were assessed and compared with data from consecutive patients undergoing initial PVI for paroxysmal AF with the cryoballoon from our specifically designed database (*RedCap Database, Nashville, Tennessee, USA)*.

### Heliostar™ RF-balloon

**Fig. 1:**
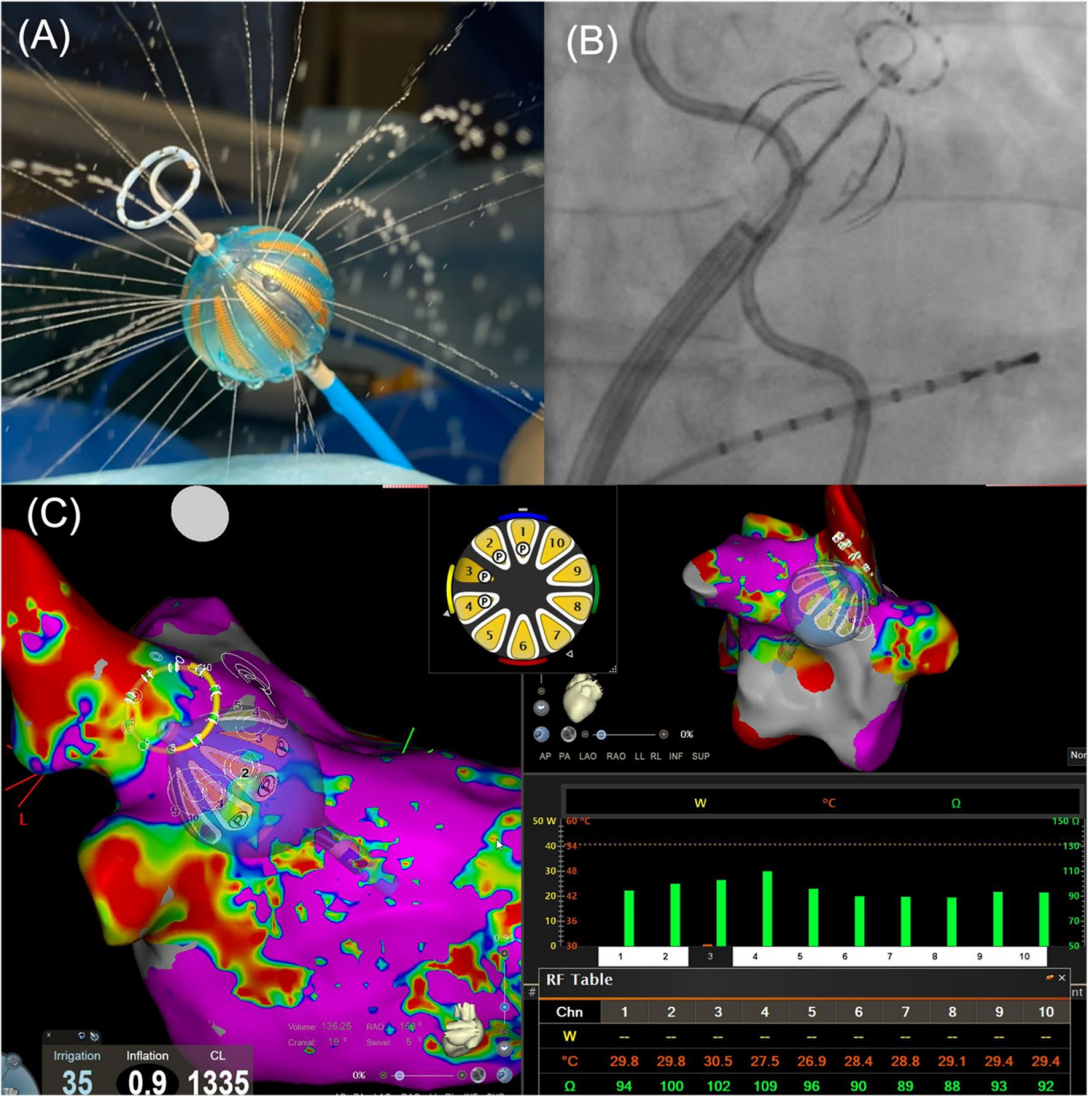
**A** Showing the 2nd generation Heliostar™ 28 mm diameter RF-balloon. **B** Fluoroscopic position of the RF-balloon at the antrum of the left superior pulmonary vein. **C** Visualization of the RF-balloon in the CARTO® 3D map indicating parameters for optimal balloon positioning. The electrode numbers highlighted in white fulfill impedance and temperature criteria for optimal balloon position

Alongside the 28 mm compliant balloon with 10 irrigated gold‐plated surface electrodes (Fig. 1), the RF-balloon ablation system comprises a 15/20 mm circular mapping catheter (Lassostar™, Biosense Webster, Diamond Bar, CA, USA), a 13.5/14Fr steerable sheath (Guidestar™, Biosense Webster, Diamond Bar, CA, USA), and a multichannel RF generator (nGEN™, Biosense Webster, Diamond Bar, CA, USA).

The first-generation RF-balloon system used a decapolar, non‐nav mapping catheter, Lassostar™ as guidewire and for EGM recording during PVI. For initial 3D map acquisition and post-PVI re-map, an additional circumferential mapping catheter (LassoNav™ Biosense Webster, Diamond Bar, CA, USA) was necessary.

The second-generation RF-balloon including the Lassostar™NAV (Biosense Webster, Diamond Bar, CA, USA) circumferential mapping catheter allowed direct 3D map acquisition via the RF-balloon making intraprocedural catheter and sheath exchange for map and re-map acquisition dispensable.

Parameters for optimal balloon positioning (Fig. 1) and ablation parameters have been previously described in detail [8, 10]. Briefly, the RF-balloon is inflated by increased irrigation flow. After balloon placement at the respective PV antrum, the ablation electrode positions can be identified in the 3D map. Anterior and posterior electrodes are defined before ablation (Fig. 1). Afterwards, energy is delivered in a unipolar mode at 15 W for 60 s anterior and 20 s posterior. All ten electrodes can be selected individually to deliver circumferential or segmental ablations up to 60 s duration. Every electrode provides real-time impedance, temperature, and electrogram data [8].

All RF-balloon procedures were performed by the same two experienced operators, and as we report our initial clinical experience, no cases had been performed prior to the present cohort.

### Cryoballoon

For cryoballoon PVI, a 28‐mm cryoballoon (Arctic Front Advance Pro™, Medtronic, Dublin, Ireland or POLARx™, Boston Scientific, Marlborough, MA, USA) was applied. The workflow of a cryoballoon PVI has been previously described in detail [4]. Briefly, the balloon was inserted through a steerable sheath (15Fr FlexCathAdvance™, Medtronic or 15.9Fr POLARSHEATH™, Boston Scientific). The cryoballoon was placed in each PV antrum under fluoroscopy guidance, and PV occlusion was verified via contrast agent application. PV potentials were recorded using a 20‐mm circular inner lumen mapping catheter with 8 electrodes (Achieve™ and Achieve Advance™ Mapping Catheters, Medtronic or POLARMAP™, Boston Scientific). The duration of cryoablation was determined by either the observed time-to-isolation (TTI; in case of visible PV signals) or achieved nadir temperature [11].

### Ablation procedures

Oral anticoagulation was discontinued the morning of the procedure and continued immediately after the procedure. All procedures were performed under deep propofol sedation amended by midazolam and fentanyl. An esophageal temperature probe (CIRCA S-CATH™, Circa Scientific, Englewood, CO, USA) was inserted transorally, and the position was confirmed with an X‐ray. An alarm was set at 38/20 °C followed by a maximal/minimal cut‐off at 39/19 °C, respectively, at which point RF delivery/cryoablation was discontinued.

After obtaining double vascular access through the right femoral vein, a decapolar catheter (Dynamic XT™, large curve, Boston Scientific, Marlborough, MA, USA) was placed in the coronary sinus (CS). Consequently, a fluoroscopy-guided single transseptal puncture using TSX™ Fixed Curve Transseptal Sheath and TSX™ Transseptal Needle (Boston Scientific, Marlborough, MA, USA) was performed. Immediately after transseptal puncture, a weight-adjusted heparin bolus was administered, with repeat activated clotting time (ACT) guided boluses every 20 min targeting an ACT of > 300 s.

For all first-generation RF-balloon procedures, the 3D map was obtained via TSX™ Fixed Curve Transseptal Sheath (Boston Scientific, Marlborough, MA, USA) and an additional 15 mm LassoNav™ (Biosense Webster, Diamond Bar, CA, USA). After map acquisition, the transseptal sheath was exchanged for the 13.5/14Fr steerable sheath (Guidestar™, Biosense Webster, Diamond Bar, CA, USA). For second-generation RF-balloon procedures, transseptal sheaths were exchanged immediately after transseptal puncture, just as for all cryoballoon procedures, and the 3D map was obtained directly via the RF-balloon and Lassostar™NAV (Biosense Webster, Diamond Bar, CA, USA).

PV signals were monitored in real‐time through the respective circumferential mapping catheter with the endpoint of an entrance block of all PVs. Finally, all PVs were re-checked for acute reconnection, and in all RF-balloon patients, a post-PVI remap was acquired confirming PV isolation via voltage map.

Phrenic nerve pacing was performed in all patients (pacing at maximal output with a cycle length of 800 ms) during ablation of the right‐sided PVs using the decapolar CS-catheter. Capture was confirmed by palpation of the diaphragmatic movement and compound motor action potential (CMAP). After sheath removal, a Z‐suture was applied for hemostasis at the femoral puncture site.

Procedure duration was defined as skin-to-skin time (groin puncture to groin suture after sheath removal), and ablation time was defined as net ablation time (start of the first ablation to end of last RF/cryoablation application). Directly, just as 2 and 24 h after the procedure, pericardial effusion was excluded using transthoracic echocardiography.

### Follow-up

For all patients, a 3- and 12-month post-ablation follow-up visit was scheduled in our outpatient clinic. Furthermore, a 6-month telephone-based follow-up was conducted. For detection of atrial arrhythmia recurrence, a 24-h Holter electrocardiogram (ECG) was performed at 3- and 12-month in-person visit and patients were requested to perform an additional 24-h Holter ECG 6 months after PVI at the attending practitioner, which was included in the telephone-based 6-month follow-up. If patients reported symptoms, an additional visit in our outpatient clinic was scheduled, and a 12-lead ECG or additional Holter ECGs were obtained. During every follow-up visit, a 12-lead ECG was recorded, and the patient’s history was taken. Arrhythmia recurrence was defined as the occurrence of any arrhythmia longer than 30 s (AF, AT, atrial flutter) after a 90-day blanking period.

### Study endpoints

Study endpoints were differences in procedural characteristics (procedure duration, ablation time, fluoroscopy use, details on RF/cryoablation applications, and isolation times) just as procedure-related complications and rates of any atrial arrhythmia recurrence (> 30 s) after a 90-day blanking period. Furthermore, a subgroup analysis within the RF-balloon group was conducted, separately comparing procedural characteristics of the second-generation RF-balloon and cryoballoon.

### Statistical analysis

Data analysis was performed using SPSS statistical software (version 26) and GraphPad Prism9. Data are shown in absolute values, percentages, and means with standard deviation. Variables were tested for normal distribution by the Shapiro–Wilk test. For the comparison of continuous variables, the Student’s *t-*test or Mann–Whitney U test were used. Categorical variables were compared using contingency tables and the application of the chi-square test or Fisher’s exact test. *P*-values < 0.05 were considered as statistically significant.

## Results

### Study population

In total, 254 consecutive patients with paroxysmal AF (63 ± 13 years, 54% male) were included between 09/21 and 11/22. Of those 30 patients underwent RF-balloon PVI whereupon in 26 patients the first-generation and in 4 patients the second-generation RF-balloon was used; the first 26 procedures were performed within the first 6 months of the study period, while the last 4 patients were treated several months later due to temporary unavailability of the device and redesign of the delivery sheath and RF-balloon. A total of 224 patients underwent cryoballoon PVI.

All patients suffered from AF symptoms (EHRA II-IV). Patient characteristics just as BMI (25.9 ± 4.9 vs. 26.9 ± 4.2; *p* = 0.3), left atrial diameter (38.0 ± 4.1 vs. 36.3 ± 5.6; *p* = 0.07), or CHA_2_DS_2_VASc-score (2.6 ± 1.1 vs. 2.2 ± 1.6; *p* = 0.1) were comparable, and there were no differences regarding comorbidities between both groups either. Baseline data of the study population is shown in Table 1.

**Table 1:** Baseline characteristics and comorbidities

Parameter	All	RF-balloon	Cryoballoon	*P*-value
Patient characteristics				
Sex (male)	137/254 (54%)	14/30 (47%)	123/224 (55%)	0.4
Age (years)	63.3 ± 13.2	67.1 ± 10.3	62.8 ± 13.5	0.08
BMI (kg/m^2^)	26.8 ± 4.3	25.9 ± 4.9	26.9 ± 4.2	0.3
CHA_2_DS_2_-VASc-Score	2.3 ± 1.5	2.6 ± 1.1	2.2 ± 1.6	0.1
LA-Diameter (mm)	36.5 ± 5.4	38.0 ± 4.1	36.3 ± 5.6	0.07
Left ventricular ejection fraction (%)	59.9 ± 5.4	58.6 ± 7.7	59.9 ± 5.4	0.7
EHRA I	0/254	0/30	0/224	
EHRA II	28/254	2/30	26/224	0.4
EHRA III	94/254	15/30	79/224	0.2
EHRA IV	132/254	13/30	119/224	0.4
Comorbidities				
Coronary artery disease	49/254	5/30	44/224	0.7
Hypertension	158/254	18/30	140/224	0.8
Diabetes	24/254	4/30	20/224	0.4
Renal failure (GFR $\le$ 50 ml/h)	18/254	4/30	14/224	0.2
Stroke	16/254	1/30	15/224	0.5
Obstructive sleep apnea	27/254	3/30	24/224	0.9

Means and standard deviation or absolute numbers and percentage are shown

### Procedural characteristics

#### RF-balloon vs. cryoballoon PVI

Acute PVI was achieved for all 896/896 PVs (100%) using the cryoballoon and for 118/120 PVs (98%) with the RF-balloon. In 2 cases, an additional single-tip catheter was necessary due to a persistent gap at the anterior carina of the left superior pulmonary vein (LSPV).

A mean of 10.7 ± 4.9 RF applications with 2.7 ± 1.2 applications per vein (LSPV 3.5 ± 2.6, LIPV 2.5 ± 2.1, RSPV 2.1 ± 1.3 and RIPV 1.9 ± 1.2) were necessary to achieve a durable PVI. Using the cryoballoon, significantly less (5.8 ± 2.2; *p* ≤ 0.001) but longer (total RF duration 584.6 ± 280.1 vs. total freeze duration 1053.8 ± 392.9; *p* ≤ 0.001) applications for the entire PVI and for each PV (Table 2) were needed. With the RF-balloon, significantly less PVs could be isolated by a single ablation (1.5 ± 1.1/4 vs. 2.7 ± 1.1/4; *p* ≤ 0.001) with a consistently lower single shot rate for each PV (Table 2). Nevertheless, the RF-balloon lead to a significantly faster isolation of the respective PV with a mean overall time-to-isolation (TTI) of 18.2 ± 7.0 s compared to 62.8 ± 35.1 s in the cryoballoon group (*p* ≤ 0.001). The TTI was significantly shorter for all PVs in the RF-balloon group (Table 2).

**Table 2:** Procedural parameters

Parameter	RF-balloon	Cryoballoon	*P*-value
PVs isolated with balloon catheter	118/120	896/896	-
RF applications/freezes per patient	10.7 ± 4.9	5.8 ± 2.2	< 0.001
RF applications/freezes per PV	2.7 ± 1.2	1.5 ± 0.5	< 0.001
*RF applications/freezes per PV*			
Left superior PV	3.5 ± 2.6	1.6 ± 1.4	< 0.001
Left inferior PV	2.5 ± 2.1	1.4 ± 0.7	< 0.001
Right superior PV	2.1 ± 1.3	1.5 ± 0.7	0.012
Right inferior PV	1.9 ± 1.2	1.4 ± 0.9	0.014
RF-ablation/freeze duration per patient	584.6 ± 280.1	1053.8 ± 392.9	< 0.001
RF-ablation/freeze duration per PV	146.2 ± 70.0	263.4 ± 98.5	< 0.001
Time-to-isolation per PV (sec.)	18.2 ± 7.0	62.8 ± 35.1	< 0.001
*Time-to-isolation per PV (sec.)*			
Left superior PV	22.0 ± 12.0	54.9 ± 39.4	< 0.001
Left inferior PV	18.3 ± 10.5	61.2 ± 46.7	< 0.001
Right superior PV	16.8 ± 10.7	62.5 ± 54.4	< 0.001
Right inferior PV	15.5 ± 9.2	59.6 ± 37.0	< 0.001
PVs isolated by single shot per patient	1.5 ± 1.1/4	2.7 ± 1.1/4	< 0.001
*Single shot isolation per PV*			
Left superior PV	5/30(17%)	147/224(66%)	< 0.001
Left inferior PV	12/30(40%)	163/224(73%)	< 0.001
Right superior PV	12/30(40%)	133/224(59%)	0.044
Right inferior PV	15/30(50%)	161/224(72%)	0.015
Fluoroscopy time (min.)	16.3 ± 7.1	11.6 ± 4.9	< 0.001
Fluoroscopy dose (mGy*cm^2^)	3792.3 ± 2678.2	1979.3 ± 1806.3	< 0.001
Contrast medium (ml)	5.1 ± 2.3	36.5 ± 10.7	< 0.001
Ablation time (min.) (first to last RF application/freeze)	43.5 ± 17.9	36.4 ± 15.6	0.08
Procedure duration (skin-to-skin, min.)	104.1 ± 35.3	69.9 ± 23.1	< 0.001
Maximum esophageal temperature (°C)	39.6 ± 2.5	-	-
Minimum esophageal temperature (°C)	-	25.4 ± 7.3	-

Means and standard deviation or absolute numbers and percentage are shown. *PV* pulmonary vein, *sec.* seconds, *min*. minutes

**Fig. 2:**
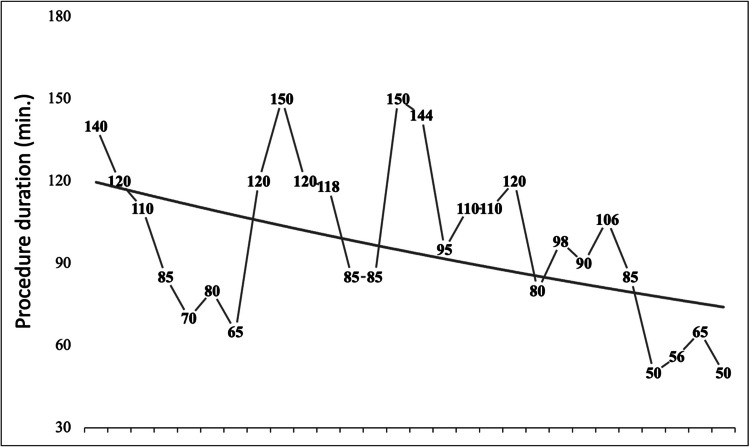
Development of overall procedure durations over time reflecting a certain learning curve, with the last ten procedures being significantly shorter. Of note, the short procedure duration of the last four procedures using the second-generation RF-balloon

While procedure duration (104.1 ± 35.3 min vs. 69.9 ± 23.1 min; *p* ≤ 0.001) and fluoroscopy time (16.3 ± 7.1. vs. 11.6 ± 4.8 min; *p* < 0.001) were longer using the RF-balloon, the net ablation time (43.5 ± 17.9 vs. 36.4 ± 15.6; *p* = 0.08) did not differ significantly. The procedure duration was liable to a learning curve (Fig. 2) with the last 10 procedures being significantly shorter (113.1 ± 30.6 min vs. 86.0 ± 38.5 min; *p* = 0.02). The mean maximum esophageal temperature was 39.6 ± 2.5 °C, and the mean minimum temperature during cryoablation was 25.4 ± 7.3 °C.

### Acute PV reconnection using the RF-balloon

Using the RF-balloon for PVI in 17/30 patients (57%), an acute PV reconnection requiring additional RF applications was observed. A detailed evaluation of PV reconnections per PV (LSPV 12/17 patients, LIPV 7/17, RSPV 4/17, and RIPV 4/17) showed that primarily the LSPV was prone to reconnection after RF-balloon ablation.

### Subgroup analysis of the 2nd-generation RF-balloon vs. cryoballoon

When comparing the small subgroup of the second-generation RF-balloon procedures with the cryoballoon PVI procedure, durations were 55.3 ± 7.1 min in the RF-balloon group vs. 69.9 ± 23.1 min in the cryoballoon group (*p* = 0.1) and fluoroscopy time was 8.6 ± 1.8 min vs. 11.6 ± 4.8 min (*p* = 0.1) with comparable RF applications/freezes for the entire PVI and every single PV, just as comparable single shot isolation rates (Table 4). The net ablation time was significantly shorter with the second-generation RF-balloon just as RF duration and TTI (Table 4).

### Complications

One pericardial effusion occurred in the RF-balloon group due to complicated transseptal access. During cryoballoon ablation in 4/223 patients (1.8%), a transient phrenic nerve palsy was observed, which resolved spontaneously in all patients within 12 months (Table 3).

**Table 3:** Complications. Absolute numbers and percentage are shown. *PV*, pulmonary vein

Parameter	All	RF-balloon	Cryoballoon	*P*-value
Pericardial effusion	1/254 (0.4%)	1/30 (3.3%)	0/224	0.1
TIA	0/254	0/30	0/224	-
Access site (major)	1/254 (0.4%)	0/30	1/224 (0.5%)	1.0
Atrialesophageal fistula	0/254	0/30	0/224	-
Phrenic nerve palsy	4/254 (1.5%)	0/30	4/224 (1.8%)	1.0
PV stenosis	0/254	0/30	0/224	-

### Outcome

A follow-up was available for all 30/30 patients undergoing RF-balloon PVI and for 214/224 patients (96%) in the cryoballoon group with a mean follow-up of 384.2 ± 58.7 days.

**Fig. 3:**
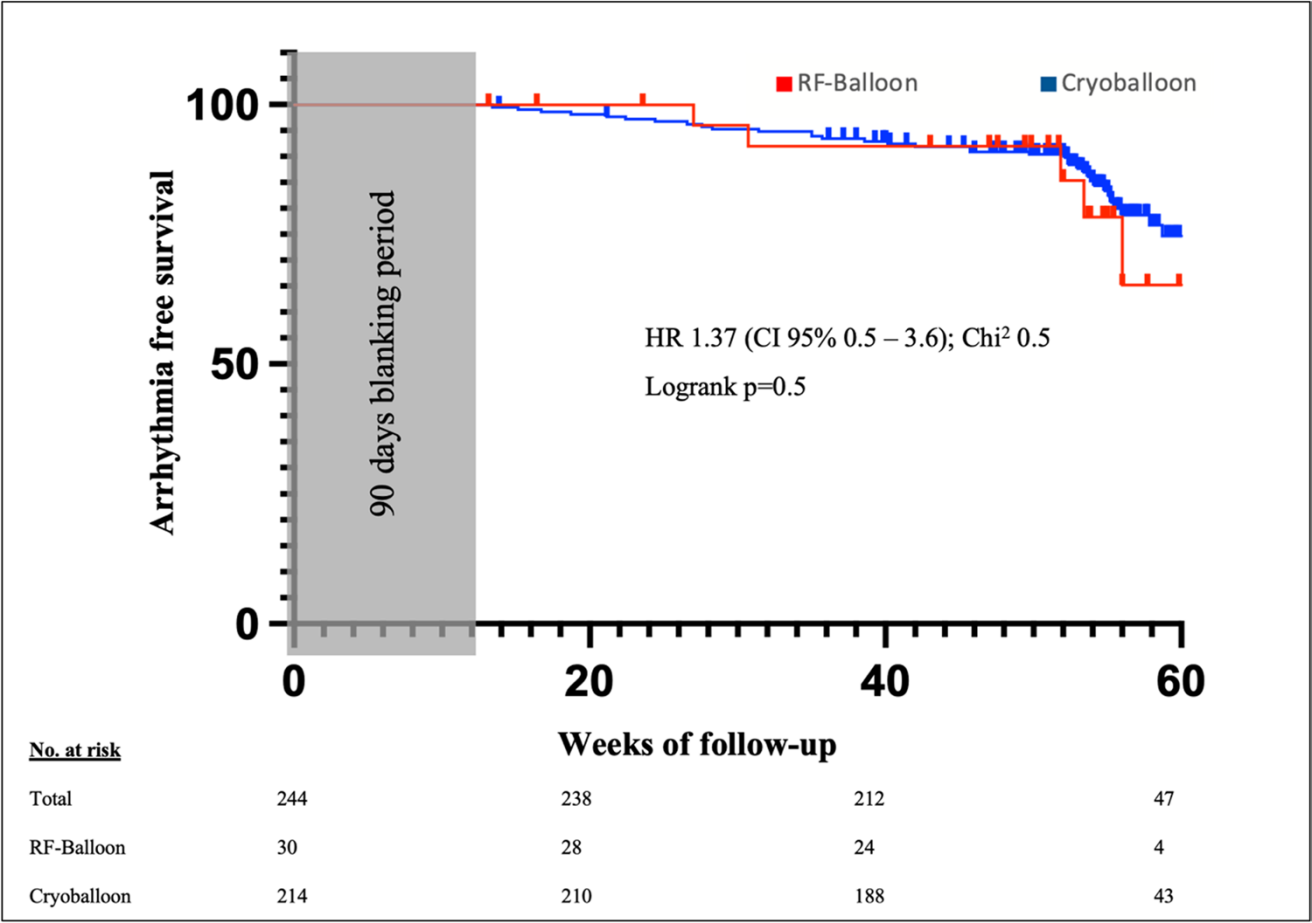
Kaplan–Meier analysis of arrhythmia-free survival after RF-balloon or cryoballoon ablation. No difference was seen between the different ablation systems (Hazard ratio 1.37, CI 95% 0.5–3.6, Chi.^2^ 0.5, logrank *p* = 0.5)

The overall freedom from atrial arrhythmia was high (Fig. 3) with 78% after RF-balloon PVI and 81% arrhythmia-free survival in the cryoballoon group and did not differ between the two ablation systems (logrank *p* = 0.5).

## Discussion

### Main findings

This single-center registry comparing two different single-shot balloon technologies for PVI provides several important findings:

1. The novel RF-balloon has shown to be safe and effective.
2. In direct comparison with the established cryoballoon, the initial application of the novel RF-balloon was associated with longer procedure durations, longer fluoroscopy times, and less single-shot PV isolations, although the net ablation time did not differ significantly.
3. Time-to-isolation was significantly shorter with the RF-balloon.
4. Of note, comparing the second-generation RF-balloon including Lassostar™NAV with the cryoballoon showed comparable procedure durations, fluoroscopy times, and single-shot PV isolations with faster TTIs and shorter net ablation time.

### Safety and acute success

No device-associated complications, phrenic nerve palsies, PV stenosis, stroke, or atrialesophageal fistula were observed using the RF-balloon. Consistent with the pilot study and the limited clinical experience available [6–10], the present data confirmed the safety of the novel RF-balloon in clinical routine use. Regarding acute success, 118 of 120 PVs were successfully isolated with the RF-balloon. In two cases, an additional single-tip catheter was applied due to a persistent gap at the anterior carina of the LSPV. Furthermore, a relatively high rate of acute LSPV reconnections was observed in this cohort requiring additional RF applications. In general, the RF-balloon has shown to be an effective tool for PV isolation. But although the balloon position at the anterior carina of the LSPV could be improved by counterclockwise rotation in the majority of patients, present data confirmed the previously published impression [10] that depending on individual PV anatomy, positioning the RF balloon against the anterior ridge may be challenging.

### Procedure characteristics

Traditionally, point-by-point radiofrequency (RF) ablation combined with 3D mapping was considered as the gold standard technology for PVI, but ultimately due to its shorter procedure durations and non-inferiority regarding safety and effectivity, the cryoballoon has emerged as one of the most frequently used techniques for de-novo PVI [4, 5, 12]. Consequently, procedural parameters of the cryoballoon PVI represent the benchmark for all new AF-ablation techniques especially for single-shot and balloon-based technologies.

### Procedure duration and fluoroscopy time

In direct comparison with cryoballoon, the initial clinical use of the RF-balloon was associated with longer overall procedure durations and increased fluoroscopy use, although a learning curve applying the new ablation tool was observed, with the last 10 procedures being significantly shorter (Fig. 2). Observed procedure durations and fluoroscopy times are consistent with the pilot study data from RADIANCE and SHINE [6, 7] but longer compared with the recently published first clinical experience [8, 9]. This difference may be explained by the underlying learning curve since Kanthasamy et al. published the results of 60 consecutive patients. When comparing the procedure durations of the last 10 patients of the present cohort with the collectives by Kanthasamy et al. and del Monte et al., procedure durations were comparable. Furthermore, in all RF-balloon patients, a detailed 3D map pre- and post-PVI was acquired, which has certainly contributed to the longer procedure durations. This was further aggravated by the necessity for an additional mapping catheter alongside with time-consuming catheter exchange using the first-generation RF-balloon without Lassostar™NAV.

### Ablation count and single shot isolation rates

The RF-balloon required a higher number of RF-ablation applications to achieve a durable PVI compared to the number of cryoablations applied during cryoballoon PVI. Although more RF applications were necessary, the overall RF duration was significantly shorter compared to cryo-application time. If an additional ablation was necessary, targeted segmental ablation could be performed at the site of the suspected gap by individual ablation electrode selection. In comparison with the published data, the present collective required overall more RF deliveries which was mostly triggered by the two cases of unsuccessful isolation of the LSPV. Moreover, the number of RF deliveries also did show a learning curve with less RF applications necessary in the last ten cases most likely due to improved RF-balloon positioning and less safety applications.

Nevertheless, the RF-ablation via a balloon catheter led to a rapid isolation of the respective PV with significantly shorter times-to-isolation (18.2 ± 7.0 s vs. 62.8 ± 35.1 s; *p* ≤ 0.001) compared with the established cryoballoon.

Although the rate of single shot isolations with the RF-balloon was comparable to the already published first clinical experience [8], the single shot isolation rates were significantly lower compared with the cryoballoon. Considering the rapid PV isolation during ablation, the higher number of RF applications necessary and the lower rate of single shot isolation may also be a dosing issue given the relatively short RF application duration of 60 s at anterior wall and 20 s at posterior wall. This certainly needs further evaluation in future studies.

### Second-generation RF-balloon vs. cryoballoon

When comparing the procedural parameters of a small subgroup of second-generation RF-balloon PVI (Table 4) with the cryoballoon, no more difference was observed regarding procedure duration or fluoroscopy time. While the net ablation time, RF-ablation duration, and time-to-isolation were significantly shorter compared to cryoablation, the number of RF applications with the second-generation RF-balloon was comparable to the number of cryoablation resulting in similar rates of single shot PV isolations. This is not only explained by a certain learning curve, but also by the availability of the Lassostar™NAV enabling 3D map acquisition directly via the RF-balloon system. Moreover, the improved balloon visualization in the CARTO® system may have contributed to these relevant performance improvements of the second-generation RF-balloon.

**Table 4:** Subgroup analysis of procedural parameters of the second-generation RF-balloon compared with the cryoballoon

Parameter	2nd gen. RF-balloon	Cryoballoon	*P-*value
PVs isolated with balloon catheter	16/16	896/896	-
RF applications/Freezes per patient	6.0 ± 1.4	5.8 ± 2.2	0.5
RF applications/Freezes per PV	1.5 ± 0.4	1.5 ± 0.5	0.5
*RF applications/freezes per PV*			
Left superior PV	2.0 ± 0.8	1.6 ± 1.4	0.09
Left inferior PV	1.3 ± 0.5	1.4 ± 0.7	0.9
Right superior PV	1.3 ± 0.5	1.5 ± 0.7	0.5
Right inferior PV	1.5 ± 0.6	1.4 ± 0.9	0.4
RF-ablation/freeze duration per patient (sec.)	348.3 ± 89.9	1053.8 ± 392.9	0.001
RF-ablation/freeze duration per PV (sec.)	87.1 ± 22.5	263.4 ± 98.5	0.001
Time-to-isolation per PV (sec.)	12.4 ± 3.5	62.8 ± 35.1	< 0.001
*Time-to-isolation per PV (sec.)*			
Left superior PV	14. ± 2.9	54.9 ± 39.4	0.001
Left inferior PV	15.3 ± .8.1	61.2 ± 46.7	0.002
Right superior PV	10.6 ± 2.2	62.5 ± 54.4	0.001
Right inferior PV	9.8 ± 2.6	59.6 ± 37.0	0.001
PVs isolated by single shot per patient	2.3 ± 1.0/4	2.7 ± 1.1/4	0.4
*Single shot isolation per PV*			
Left superior PV	1/4 (25%)	147/224(66%)	0.1
Left inferior PV	3/4 (75%)	163/224(73%)	1.0
Right superior PV	3/4 (75%)	133/224(59%)	0.7
Right inferior PV	2/4 (50%)	161/224(72%)	0.3
Fluoroscopy time (min.)	8.6 ± 1.8	11.6 ± 4.9	0.1
Fluoroscopy dose (mGy*cm^2^)	1811.8 ± 542.9	1979.3 ± 1806.3	0.6
Ablation time (min.) (first to last RF application/freeze)	23.3 ± 5.4	36.4 ± 15.6	0.02
Procedure duration (skin-to-skin, min.)	55.3 ± 7.1	69.9 ± 23.1	0.1

Means and standard deviation or absolute numbers and percentage are shown

*PV* pulmonary vein, *sec*. seconds, *min*. minutes

### Outcome

Regarding freedom from atrial arrhythmia, no difference was seen between both systems. After a median follow-up of 12.5 months, 78% and 81% of patients with PAF were in stable sinus rhythm which is consistent with previously published data for cryoballoon PVI in patients with PAF [4] and the very limited follow-up data available for the RF-balloon from the pilot study [6].

## Limitations

The present study is a single-center, nonrandomized observational study with all its inherent limitations. To reduce selection bias, we included consecutive patients, and there were no significant differences in baseline data regarding patient characteristics and comorbidities. The RF-balloon group, and especially the patients treated with the 2nd generation RF-balloon, comprised a small sample size, with its inherent statistical limitations. But, considering the novelty of the device, the relevant adaptations in the balloon design, and the overall low number of patients treated with the RF-balloon by now, also small sample sizes are of clinical interest. Given the observed learning curve, the above-mentioned differences between both systems potentially would have decreased over time with a larger sample size. Furthermore, all follow-up data were obtained using medical history and 24-h Holter, so asymptomatic episodes of AF have potentially been missed in the absence of continuous rhythm monitoring. Certainly, present findings, derived from the initial clinical evaluation during the limited marked release, need further randomized controlled verification in the future.

## Conclusion

The novel multi-electrode RF-balloon has shown to be safe and effective with good long-term results. In comparison with the cryoballoon, time-to-isolation was significantly shorter, but procedure durations were longer, and fluoroscopy exposition was higher using the first generation Heliostar™ without LassostarNav®. This can be attributed to a learning curve applying a new ablation system and the necessity for separate 3D map preparation. Considering the encouraging results with the second-generation RF-balloon, more experience is needed in the future to determine the potential benefits.
